# HIV-Associated Cancer Biomarkers: A Requirement for Early Diagnosis

**DOI:** 10.3390/ijms22158127

**Published:** 2021-07-29

**Authors:** Zodwa Dlamini, Mzwandile Mbele, Tshepiso J. Makhafola, Rodney Hull, Rahaba Marima

**Affiliations:** SAMRC Precision Oncology Research Unit (PORU), Pan African Cancer Research Institute (PACRI), University of Pretoria, Hatfield 0028, South Africa; zwai2002@hotmail.com (M.M.); jmakhafola@cut.ac.za (T.J.M.); rodneyhull@gmail.com (R.H.); rahaba.marima@up.ac.za (R.M.)

**Keywords:** cancer, HIV/AIDS, biomarkers, diagnosis, prognosis, HAART

## Abstract

Globally, HIV/AIDS and cancer are increasingly public health problems and continue to exist as comorbidities. The sub-Saharan African region has the largest number of HIV infections. Malignancies previously associated with HIV/AIDS, also known as the AIDS-defining cancers (ADCs) have been documented to decrease, while the non-AIDS defining cancer (NADCs) are on the rise. On the other hand, cancer is a highly heterogeneous disease and precision oncology as the most effective cancer therapy is gaining attraction. Among HIV-infected individuals, the increased risk for developing cancer is due to the immune system of the patient being suppressed, frequent coinfection with oncogenic viruses and an increase in risky behavior such as poor lifestyle. The core of personalised medicine for cancer depends on the discovery and the development of biomarkers. Biomarkers are specific and highly sensitive markers that reveal information that aid in leading to the diagnosis, prognosis and therapy of the disease. This review focuses mainly on the risk assessment, diagnostic, prognostic and therapeutic role of various cancer biomarkers in HIV-positive patients. A careful selection of sensitive and specific HIV-associated cancer biomarkers is required to identify patients at most risk of tumour development, thus improving the diagnosis and prognosis of the disease.

## 1. Introduction

Cancer is a genetically and clinically diverse disease that leads to an uncontrolled proliferation of abnormal cells due to the inhibition of apoptotic processes. Its pathogenesis, metastatic potential, aggressiveness, and response to treatment is known to be different among individual patients [1,2,3]. The role of genetic factors has been suggested in cancer pathogenesis since variation has been marked in individuals with the same type of cancer. Globally, the coexistence of HIV/AIDS and cancer, particularly the non-AIDS-defining cancers (NADCs) is growing. This is particularly observed in the sub-Saharan African region with the highest HIV/AIDS infections [4]. In the US, cancer risk among HIV-positive people was also observed [5]. Among these HIV-infected individuals, cancer risk has increased because of immunosuppression, frequent coinfection with oncogenic viruses and risk behavior including poor lifestyle [2,3,6]. Following the advent of the highly active antiretroviral treatment (HAART) in 1996, the health-related quality of life (HRQoL) for HIV-positive patients has significantly improved [7,8,9]. This has resulted in an increase in the number of individuals living with HIV and the aging HIV-positive population [5,10,11]. Reports revealing the association between HIV/AIDS and tumourigenesis are rapidly emerging. This pathogenesis could be attributed to various factors. These may include the viral (HIV) factors, immunosuppression, coinfection with oncogenic viruses, HAART components and poor lifestyle [1,12]. These risks are known to be high for malignancies caused by viral infections and include the AIDS-defining cancers (ADCs) such as Kaposi’s sarcoma, Non-Hodgkin lymphoma (NHL) and invasive cervical cancer [1]. Compared to the general population, HIV-positive people have an increased propensity to develop malignancy [13]. 

**Figure 1:**
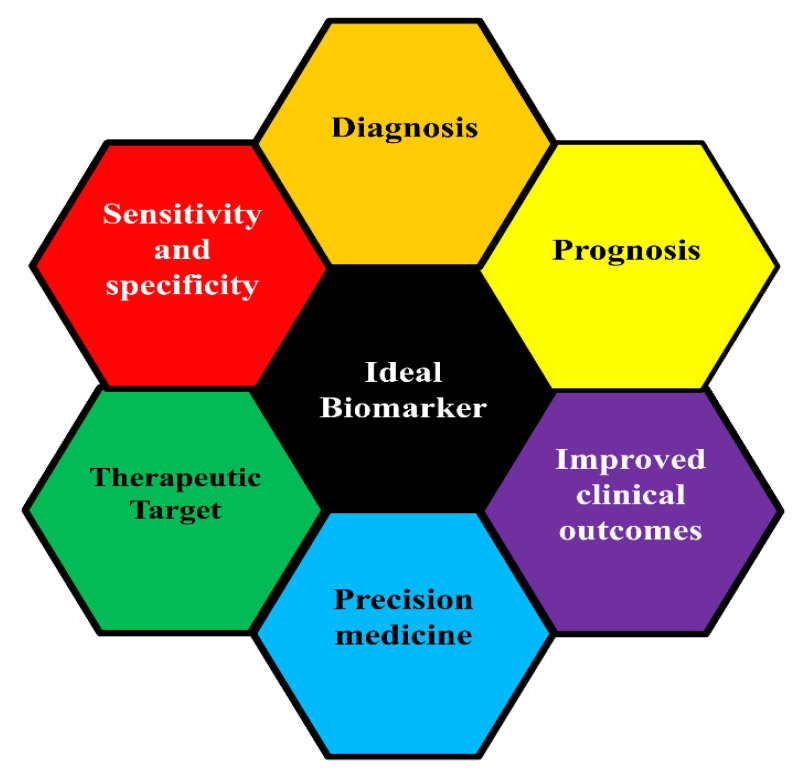
Ideal biomarkers are key to improved patient outcome. Biomarkers can be targeted for improved diagnosis, prognosis, and therapeutics. The identification of ideal biomarkers holds key potential to personalised medicine and overall improved clinical outcome.

As defined by the US National Cancer Institute (NCI), a biomarker is a biological molecule found in body fluids such as blood, or can be found in tissues, and is a sign of normal or abnormal process, or disease or condition (WHO, 2003). The World Health Organisation defines a biomarker as process that can be measured, or a substance or a structure that can influence or predict the outcome or disease (WHO, 2001). In medicine, biomarkers are useful in screening, diagnosis, prognosis and treatment purposes. There are different types of biomarkers, some of which overlap. Such examples may include gene/protein-based biomarkers [1,14,15]. Early detection of cancer biomarkers in HIV-positive individuals would be useful to identify patients at high risk of tumour development. Cancer biomarkers are discovered through using molecular and cellular methodologies that focus on disease and drug mechanisms. These indicate the interaction of novel therapies with their intended target pathogenesis of the disease itself. Biomarkers consist of genomic and proteomic patterns, chromosomal abnormalities, epigenetic signatures, single genes or proteins. This causes biomarkers to play a significant role in cancer screening, early diagnosis, cancer stratification, efficacy prediction and adverse reactions. The application of precision medicine in the management of cancer patients is largely attributed to biomarkers. The core of personalised medicine for cancer depends on the discovery and the development of biomarkers. The importance of molecular biomarkers is based on the sensitivity, specificity and predictability (Figure 1). This information is required to facilitate the development of improved classification, which will aid clinical outcomes and reduce therapeutic instability [16,17]. With the rising cancer cases in HIV-positive patients, particularly the NADCs, the need to develop biomarkers is profound. To the best of our knowledge, there are no molecular biomarkers currently with widely proven utility for predicting clinical outcomes, although some biomarkers are promising. This review will primarily focus on the risk assessment, diagnostic, prognostic and therapeutic roles of various cancer biomarkers in HIV-positive patients.

## 2. Cancer Prevalence in HIV-Positive Patients 

The spectrum of cancer types seen in HIV-infected individuals encompasses a number of NADCs [18,19]. These include cancers of the lung, breast, prostate, liver, throat, anus, Hodgkin lymphoma and non-melanoma skin cancers [4,20,21]. The incidence of cancer has shown to account for a quarter amongst HIV-infected individuals’ deaths and indicates to surpass AIDS as the leading mortality of HIV-infected individuals in high-income countries [19,22]. In sub-Saharan African, the incidence of NADCs is on the rise [4]. The incidence rate of many non-AIDS-defining cancers has indicated a risk of increase, while the incidence of AIDS-defining cancers such as Kaposi’s sarcoma (KS) and Non-Hodgkin lymphomas (NHL) has been reduced by the use of antiretroviral therapy (ART) [11,23]. The impaired immune function and high prevalence of non-HIV cancer risk factors contribute to the overall burden of cancer. Park et al., (2016) have reported that half the number of people living with HIV/AIDS (PLWHA) are smokers with 2.5 times higher risk compared to USA adults [24]. This study also reported that one quarter of PLWHA had the hepatitis C virus (HCV) infection and 5% were infected with the hepatitis B virus (HBV). When compared with adults in the US, the HCV infection in PLWHA was 12–40 times higher and HBV was 10–25 times higher [24,25]. The sub-Saharan African (SSA) region, particularly South Africa, has 32 cases per 100,000 women, the highest age-standardised incidence of cervical cancer globally [26]. 

## 3. The Genetics of Molecular Biomarkers

**Figure 2:**
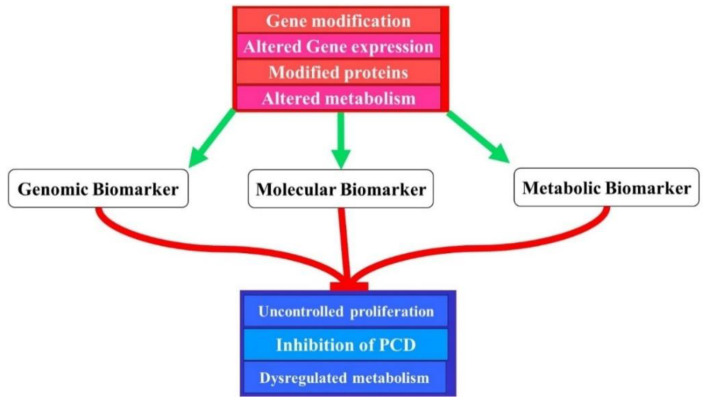
Different biomarkers may be identified as a result of various genetic and proteomic alterations. These alterations may lead to different pathologic conditions such as cancer by breaching proliferation marks, invading programmed cell death mechanisms and altering metabolism.

Biomarkers are proteins that have several cellular functions and are vital in regulating these functions. These proteins are encoded by genes that are located in chromosomes, and each gene has its particular location in the chromosome. The development of cancer can be caused by the alteration of these genes. The alteration may be disease causing or modify the pathogenesis and progression of the disease. Disease-causing alterations are called mutation and forms as a result of deletion/insertion in one or more nucleotides or substitution of nucleotides. Such mutations have been identified in a number of cancer types resulting from DNA screening. Figure 2 shows how different alterations in gene and protein sequence and expression can lead to identifying different biomarkers. The mechanisms of tumour genesis, development and response to therapy can be demonstrated through biomarkers. The genetics of biomarkers may illustrate the molecular alterations in nucleic acid that may lead to defects in structure or regulation of the cell. Biomarkers require clinical relevance with a potential to offer information that will provide understanding of a disease or to enhance treatment modalities [16,27]. 

### 3.1. Classification of Cancer Biomarker

Biomarkers are classified based on certain parameters such as functions and characteristics such as Type 0, Type I and Type II [28,29,30]. Type 0 biomarkers are used to measure the natural history of diseases. These biomarkers are associated with known clinical indicators. Type I biomarkers correlate with the efficacy of pharmacologic agents. Type II biomarkers are surrogate endpoint biomarkers that are intended to substitute for clinical endpoints [29,31]. Recently, tumour biomarkers are grouped into certain categories such as proteins, glycoproteins, hormones, receptors, oncofetal antigens, genetic markers and RNA molecules [9]. Cancer biomarkers are also known to be classified into diagnostic, predictive, prognostic and pharmacodynamic biomarkers [27,32,33,34,35]. Prediction biomarkers, also known as response markers, are used to assess the effect of a specific drug to allow the clinicians to select chemotherapeutic agents which will have the best positive response on the patient [35,36]. Prognostic biomarkers are used to analyse the overall outcome of the disease [37,38]. Lastly, pharmacodynamic biomarkers are utilised to select chemotherapeutic agents’ doses in a given set of tumour–patient conditions. These biomarkers are also used to assess the impending treatment effects of a drug. During cancer development, the diagnostic markers may be present at any stage [8,39].

### 3.2. Biomarkers Used in HIV-Associated Cancer Diagnosis and Prognosis

Compared to the broad spectrum of the NADCs, ADCs that include Kaposi’s Sarcoma, cervical cancer and NHL are on the decline since the introduction of HAART. The diagnostic and prognostic challenges of these HIV-associated cancers are discussed below [21]. 

#### 3.2.1. Diagnosis and Prognosis Challenges of HIV-Associated Cervical Cancer

Cervical cancer is a disease that develops as a result of a persistent infection with high-risk human papillomavirus (hrHPV) types, resulting in premalignant precursor lesions also known as cervical intraepithelial neoplasia (CIN) [34,40]. Human papilloma virus (HPV) is defined as the cause of cervical cancer, although only 2% of cervical HPV are known to result in cervical cancer [41]. Cervical cancers have two common histologic types, namely, squamous cell carcinoma (SCC), accounting for 70% of all adenocarcinomas [26,42]. In 2018, new diagnosed cervical cancer cases worldwide were ~569,000. Furthermore, ~311,000 deaths were associated with cervical cancer. The low-to-middle income countries (LMICs) such as South Africa accounted for ~90% of these deaths [26]. Abnormalities in cellular proliferation, maturation and nuclear atypia are characteristics of CIN [43]. According to Flepitsi et al. (2014), CIN may regress to normal or progress to invasive cervical cancer if untreated [1]. It is reported that approximately one-third to one-half of the CIN I and CIN II cases regress without treatment; including when the abnormality of the lesions is more severe, and they are less likely to regress [1]. Grading of CIN lesions is vital for clinical management of patients and specific biomarkers are required for grading and accurate diagnosis. The inaccurate grading results in inaccurate diagnosis and, therefore, ineffective treatment of CIN.

A whole host of novel biomarkers for the diagnosis of cervical cancer have been identified. These include the presence of HPV E6/E7 mRNA, miR-9 and patterns of DNA methylation. Protein expression biomarkers include p16INK4a/ki-67, SCC-Ag, M-CSF and VEGF. However, not all these biomarkers are suitable for the diagnosis of HIV-associated cervical cancer [44,45,46].

Cervical cancer diagnosis is achieved by the detection of HPV DNA in cervical tumour cells, which has proven to be a good diagnostic and risk predictor tool (Table 1) [47]. The initiation and mediation of the oncogenic process of cervical cancer occurs by the upregulation of HPV E6/E7 oncoproteins. The overexpression of these oncoproteins serves as a biomarker for increased cervical cancer risk [48,49,50]. The hrHPV 16 and 18 subtypes have a vital role in malignant transformation of cells by developing E6 and E7 viral regulatory proteins [25]. The viral regulatory proteins, E6 and E7, are involved in cell proliferation and survival. The microRNA miRNA9 has been shown to be an accurate prognostic and diagnostic biomarker for cervical cancer [51]. This miRNA is known to play a role in neurogenesis, which is a process that also plays a role in the progression of many cancers [51]. Changes in DNA methylation is a major epigenetic mechanism that regulates gene expression, genomic imprinting, cell differentiation, development and inflammation [52]. These epigenetic changes also play a role in the early diagnosis of cervical cancer.

The most vital biomarkers implicated in cervical cancer are HPV and oncogene E6 and E7 [49]. Another protein, the Ki-67 is the cell proliferation biomarker which plays an important role in confirming the diagnosis and CIN grading [53]. This biomarker is known to detect a nuclear antigen found only in cell proliferation but not in other cells [54]. Furthermore, Ki-67 is known to be more intensely stained in HPV-positive than HPV-negative epithelium. The p16 protein, a cyclin-dependent kinase (CDK) inhibitor has a specific biomarker functions used to identify squamous and glandular dysplastic cervical epithelium. In cervical epithelial cells that are transformed due to the hrHPV E7 oncoprotein expression, the overexpression of p16 has been observed [40]. In a study performed by Carozzi and colleagues (2013), p16 has been shown to be a biomarker for CIN II or for the development of CIN II within 3 years in HPV-positive women [55]. It has been reported that p16 alternatively complements Ki-67 for HPV-related neoplasia [50,56,57]. Ki67 and p16 are better used in combination that alone, in the diagnosis of cervical cancer. Cytokeratin (CK) 17, a biomarker for endocervical reserve stem cells, plays an important role in the differentiation between immature squamous metaplasia and high-grade CIN III. CK-17 is a biomarker that is not expressed in cervical glandular epithelial cells, squamous cells, or mature squamous metaplastic cells. However, they are known to be specific for immature metaplastic cells and reserve cells (Mockler et al., 2017) [57,58]. The important tumour suppressor protein p53 is a known nuclear phosphoprotein encoded by the p53 gene and responsible for cell proliferation and apoptosis control. Alterations in the p53 gene are closely associated with invasive cancers as a result of loss of tumour suppressor function. The overexpression of p53 biomarker have been shown in cervical cancers [59,60]. Table 1 outlines HIV-associated cancer biomarkers.

The measurement of serum levels of squamous cell carcinoma antigen (SCC-Ag), which is a serine protease inhibitor (Serpin), is a good indicator of the presence of cervical cancer, or additionally, as a prognostic indicator. SCC-Ag levels are elevated in cervical cancer [61]. The macrophage colony-stimulating factor (M-CSF) is a hematopoietic growth factor and can serve as a biomarker in multiple cancers [61]. The vascular endothelial growth factor is used as a diagnostic biomarker in not only cervical, cancer but also in breast and endometrial cancer [62].

**Table 1:** Identified biomarkers and the related changes in HIV-associated cervical cancer.

HIV-Associated Cervical Cancer	Changes in HIV-Cervical Cancer	References
HPV DNA	Elevated	[40,63]
HPVE6/E7	Elevated	[47,50,64]
Ki-67	Elevated	[65,66]
P16	Elevated	[40,65]
CK17	Elevated	[40,67]
MCM	Elevated	[68,69]
CDC6	Elevated	[70,71]
Ribosomal protein S12	Elevated	[72]
P53	Elevated	[43,73]
PCNA	Elevated	[74,75]
MIB-1	Elevated	[75]
P63	Suppressed	[40,76]
CD44	Elevated	[77]

#### 3.2.2. Diagnosis/Prognosis Challenges of HIV-Associated Non-Hodgkin Lymphoma

NHL is reported as the second most common malignancy in HIV-infected patients and is characterised by diffused large B-cell lymphoma (DLBCL) [17]. NHL cases are believed to arise from B-cell progenitors and develop into various entities that are grouped into three, the low, intermediate and high-grade NHL [78]. Heterogeneous diseases such as DLBCL differ in genetic abnormality, morphology nature and clinical features, and patients vary in prognosis and respond differently to treatment [79,80]. DLBCL develops from normal antigen-exposed B-cells that have moved to or through germinal centres [34,80]. There are two subgroups identified by gene expression profiling: (i) germinal centre B-cell-like (GCB) lymphomas (typically CD10+ and BCL6+), and (ii) non-GCB lymphomas that are developed from cells resembling activated B-cell-like lymphomas [27,33,81,82]. Patients with GCB DLBCL display a better progression and overall survival than patients with non-GCB DLBCL, regardless of the international prognostic index score (IPI) [34,83,84]. IPI is a useful clinical tool that aids in prognostic prediction of patients with aggressive NHL [85]. It has been also suggested that the sub-classifications of DLBCL into GCB and non-GCB may be a vital prognostic factor.

The quality and quantity of affected lymph nodes for assessing morphology and architecture are the first requirement in the diagnosis of NHL [86,87,88]. Blood count, differentiation of white blood cells, count of platelet and examination of peripheral smear for the presence of atypical cells are performed to detect the involvement of peripheral blood and bone marrow [89]. These tests are followed by pathological tests such flow cytometry or immunohistochemically staining for immunophenotype [89]; Ki-67 or MIB-1 staining (an antibody against Ki-67) are used to identify aggressive lymphomas as these may be indicating a high growth fraction of tumours [89]. Ki-67 expression in DLBCL patients is associated with poor outcome and survival [11,90,91]. B-cell biomarkers such as CD19, CD20, CD22, CD79a and PAX-5 that play an important role in immunophenotypic expression patterns of DLBCL and flow cytometry have shown surface immunoglobulin light chain restriction in a majority of cases (Table 2) [34,92]. Since PAX-5 is a B-cell restricted transcription factor, positive PAX-5 immunostaining indicates a strong association with B-cell differentiation [34], while positive staining of biomarkers such as CD10, bcl-6 and MUM-1 distinguish GCB from non-GCB DLBCL [1,34]. Fork box protein P1 (FOXP1) is a transcriptional regulator of the B-cell development and has been found to be overexpressed in non-GCB DLBCL than in GCB DLBCL [88,92,93]. The poor survival and prognosis have been associated with FOXP1 [94]. For this reason, it has been recommended that FOXP1 should be used to distinguish non-GCB from GCB DLBCL to improve the diagnosis and predict prognosis of DLBCL.

In some studies, fluorescence in situ hybridisation (FISH) analysis of cMYC, a transcription factor that functions in regulating cell growth and cell cycle, has shown to occur in 10–15% of DLBCL lymphomas and is associated with worse prognosis outcomes [95]. Furthermore, the translocations of MYC confers poor prognosis in patients treated with cyclophosphamide, hydroxydaunorubicin, oncovin and prednisone (CHOP) regime [1]. Modified immune mechanisms play a critical role in the pathogenesis of NHL. Increased prevalence of NHL has been reported among HIV-positive patients, patients with autoimmune disease and transplant recipients [23,36]. B-cell activation is commonly shown in HIV infection, which is caused by the overproduction of B-cell stimulatory cytokines, such as IL-6 and IL-10. This also applies for the stimulation of B-cells by HIV and other microbial antigens [81,91]. HIV also induces the production of inflammatory cytokines that cause B-cell stimulation, activation and proliferation. Cell lines derived from HIV-NHL show the expression of cytokines including interleukin 6, 10 and tumour necrosis factor-α [96,97,98,99]. B-cell activation is characterised by the proliferation lymphocyte, class switch recombination (CSR) and somatic hyper-mutation, all of which are prone to result in DNA replication errors that may lead to lymphomagenesis. Table 2 outlines HIV-associated NHL biomarkers. 

**Table 2:** Identified biomarkers and the related changes in HIV-associated Non-Hodgkin lymphoma.

HIV-Associated NHL Biomarkers	Changes in HIV-NHL	References
LDH	Elevated	[93,100,101]
Ki-67/MIB-1	Elevated	[102,103]
CD19, CD20, CD22	Elevated	[79,104]
PAX-5	Elevated	[79,104]
CD10	Elevated	[17,102]
bcl6	Elevated	[1,105]
MUM-1	Elevated	[17,102]
cMYC	Elevated	[106,107]
IL-6	Elevated	[39,108]
IL-10	Elevated	[39,108,109]
TNF-α	Elevated	[39,110]
CRP	Elevated	[93,108]
sCD23, sCD27, sCD30, sCD44	Elevated	[92,94]
EBV DNA	Elevated	[95]
CXCL13	Elevated	[39,94]
FLC	Elevated	[39,111]
FOXP1	Elevated	[1,112]
B2M	Elevated	[1,100]

#### 3.2.3. Diagnosis/Prognosis Challenges of HIV-Associated Kaposi’s Sarcoma

KS is defined as an endothelial neoplasia that is located in cutaneous lesions and is known as a common malignancy in HIV patients [28]. HIV-associated KS (HIV-KS) is reported as a low-grade vascular tumour which is associated with human herpesvirus-8 (HHV8)/KS-associated herpes virus (KSHV) infection and is the most frequent and aggressive type [35,78]. The primary target for KS involves the skin [36,113]. Multiple mucocutaneous lesions from early or patch stage into plague stage and then tumour or nodular stage contain spindle-shaped tumour cells. KS has a variable clinical course, and this can pose challenges in histologic diagnosis [32]. KS differs in characteristic features from other benign or malignant vascular tumours and other nonvascular spindle tissue neoplasms. This is a vital challenge and require great investigations [33]. In its early stages, lesions may either regress or progress. Progression represents the expression of HHV8 latency that include latent nuclear antigen-1 (LANA-1) [28], cyclin-D1 [34,35] and bcl-2 (Table 3) [36]. Receptor tyrosine c-kit gene expression profiling in cultured endothelial cells has a functional role in KS tumourigenesis-activated HHV8-related induction [37,38]. Table 3 outlines HIV-associated KS biomarkers. 

There are eight variable diagnoses of KS. These include cutaneous angiosarcoma, spindle cell haemangioma, pyogenic granuloma and spindled melanoma. vascular transformation of lymph nodes, dermatofibrosarcoma protuberans, pilar leiomyoma and stasis dermatitis [33]. KS histology indicates the progressive proliferation of spindle-shaped cells which are associated with KSHV/HHV8 [39]. The immunohistochemical detection of HHV8 of fixed tissues might be a diagnostic tool to differentiate KS. HHV8 encodes for numerous proteins used to induce or maintain KS lesions, such as K12, K13/viral FADD-like interferon converting enzyme inhibitory protein (vFLIP), vCyclin and the LANA-1 that is required for cellular transcription [40,41,42]. In the viral genome, the open reading frame encodes for the HHV8 LANA-1 protein which reveals its expression during viral latency and its functional role in viral integration into the host genome. The HHV8 LANA-1 protein has been reported to have an interference involvement in apoptosis through interactions with p53 [43]. The antibodies such as platelet/endothelial cell adhesion molecules, PECAM1 (D2-40, CD31), a hematopoietic progenitor cell surface protein and Friend leukaemia virus integration 1 are used in immunohistochemical staining to distinguish cutaneous KS from other diseases [31,49].

There are a number of peptide growth factors of HIV that encode Tat protein, inflammatory cytokines and KSHV/HHV8 gene products involved in KS cell growth and development [55]. The antigens for HHV8 affect cell signaling pathways and deregulate immune response and apoptosis through vCyclin, vFLIP, bcl2 oncogene, viral interferon regulatory factor and vIL-6 [1]. The mutations in immune cells may play a vital function in the neoplastic process [40,137]. Immune activation has a cooperative function with growth factors and HIV-1 Tat protein in KS development [65]. HIV-KS cells produce cytokines and angiogenic growth factors such as fibroblast growth factors (FGFs), tumour necrosis factor-α (TNF-α), interleukin-1 (IL-1), IL-6, Tat and oncostatin M. They express high affinity receptors for some cytokines [66,67]. 

## 4. HIV-Associated Cancer Mechanisms

**Figure 3:**
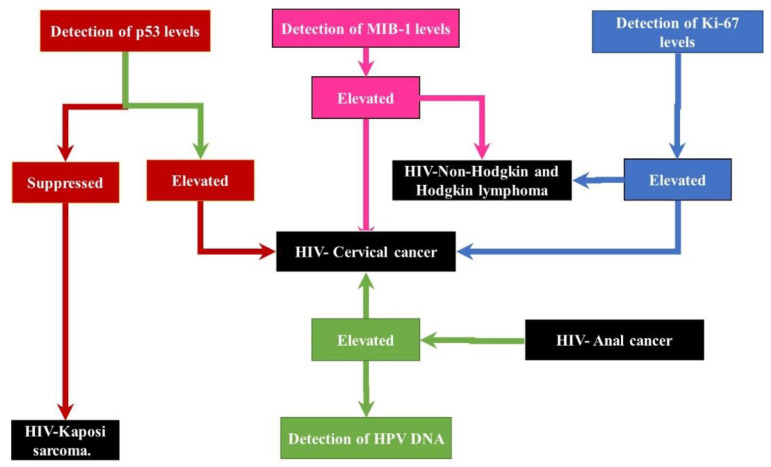
Diagnostic scheme for some HIV-associated cancers using specific biomarkers. The above diagram represents the use of the expression levels of p53, Ki-87 and MIB-1 proteins as well as the presence of HPV-DNA as diagnostic biomarkers. The expression levels of p53 can be used to diagnose Kaposi’s sarcoma (suppressed) and cervical cancer (elevated) [43,125]. The diagnosis of cervical cancer can be confirmed by the increased presence of HPV DNA as well as elevated levels of Ki-67 and MIB-1. While elevated levels of KI-76 and MIB-1 with no effects on p53 or presence of HPV DNA can serve as diagnostic markers for Non-Hodgkin lymphoma, the presence of HPV DNA without the increase of p53, Ki-67 or MIB-1 is indicative of anal cancer [34,63,139].

The biological mechanisms of all diseases are based on the molecular analysis at the protein, DNA, RNA and mRNA levels that can contribute to identifying tumour subtypes. Each of the subtypes will have a unique prognostic outcome and/or response to treatment [19]. Biomarkers are the targets to categorise patient populations in order to make it possible for drugs to reach the intended targets. The most valuable biomarkers must be highly sensitive, specific, reproducible and predictable [23,24]. These biomarkers are altered proteins in vital pathways that are involved in these diseases. Some biomarkers may not be specific in one HIV-associated cancer and may overlap with other cancers (Table 4). This requires thorough screening and investigation in order to eliminate inaccurate diagnosis. The overlapping of biomarkers is possible since similar molecular pathways may be shared. This can be eliminated by selecting sensitive and specific biomarkers focused on HIV-associated cancers. Figure 3 shows an example of the use of various biomarkers to diagnose specific HIV-associated cancers. Table 4 and Table 5 illustrate the diagnostic use of biomarkers. The characterisation of the different expression profiles of various biomarkers can be used to stratify cancers by stage in a molecular fashion. This is illustrated for cervical cancer in Table 4. The expression levels of Ki67 and p16 can be used individually and in combination to diagnose cervical cancer. When used alone, Ki67 has higher sensitivity but lower specificity compared to p16, which has lower diagnostic sensitivity but higher specificity. When used together in a single diagnostic test, based on Ki67 and p16 expression levels, a more specific and sensitive diagnostic tool can be created [138].

Biomarkers may be classified into different types based on their function’s characteristics. Type zero biomarkers measure and correlate with the disease history. Type I biomarkers correlate with the pharmacological agents’ effectiveness, while type II can be used as clinical endpoints substitutes [25]. Sometimes biomarkers are used in combination and not individually, such as Ki67 and p16 to enhance cervical cancer diagnosis (Table 5). 

## 5. Treatment for HIV-Associated Cancers

HAART use has shown a considerable reduction in the incidence of HIV-associated cancers and these results were mostly noted in Kaposi’s sarcoma and Non-Hodgkin lymphoma [147]. Looking at the HAART era, the incidence and proportional mortality for certain non-AIDs defining malignancies (NADCs) have experienced an increase, and that includes lung cancer [1,78,106]. The increase in incidence is attributed to longer life expectancy due to HAART. The relationship between the use of HAART components and tumourigenesis may be unfolding, but still remains to be fully comprehended [147]. 

Cancer research has reached a molecular age and characterises tumours with respect to causation by oncogenic virus. Features related to immunophenotype and genotype are also important in optimising treatment [75,148]. In HIV-infected individuals for which tumour biology has indicated variation from that of non-infected individuals, genomic characterisations have been applied [14,23,78]. The tumour biology may vary amongst HIV-positive patients. This may be due to contributing factors such as HIV replication, HAART use, aging, immunosuppression and poor lifestyle. Such cases vary between high- and low-income countries, as low-to-middle-income countries have the leading HIV-associated cancer cases. Given that, it clearly shows that treatment of HIV-associated cancer is still challenging and requires more research [4,149]. With the rising cases of NADCs, particularly in the sub-Saharan African Region, and common use of traditional herbal medicine, exploring medicinal plants for anti-cancer use may hold promising potential to treat HIV-associated cancers.

## 6. Conclusions

HIV-associated cancers are a public health problem, as both HIV and cancer emerge as colliding morbidities. Invasive cervical cancer is also a challenge in HIV-positive women. Contributing factors, which may include the use of HAART components, cannot be ignored. The variability in tumour biology between the HIV-positive patients also poses as a challenge. A careful selection of sensitive and specific HIV-associated cancer biomarkers is required in order to improve the diagnosis and prognosis of these comorbidities. Precision medicine holds promising potential in the fight against these colliding pandemics, and the identification of unique and common HIV-associated biomarkers will aid in improving the overall patient outcome.

## Figures and Tables

**Table 3:** Identified biomarkers and the related changes in HIV-associated Kaposi’s Sarcoma.

HIV-Associated KS Biomarkers	Changes in HIV-KS	References
HHV8/LANA-1	Elevated	[1,114,115,116]
Cyclin D1	Elevated	[114,117,118]
bcl2	Elevated	[96,97,99]
c-kit	Elevated	[98,119]
K12	Elevated	[25,120]
K13/vFLIP	Elevated	[25,121,122]
vCyclin	Elevated	[97,123,124]
P53	Suppressed	[125,126]
pRb	Suppressed	[25,117]
D2–40	Elevated	[35,115,127]
CD31	Elevated	[116,128]
CD34	Elevated	[128,129,130]
FLI1	Elevated	[1,129]
vIL-6	Elevated	[25,131,132]
Tat	Elevated	[92]
bFGF	Elevated	[133]
TNF-α	Elevated	[1,133]
IL-1	Elevated	[134]
Oncostatin M	Elevated	[133,135,136]

**Table 4:** Biomarkers used to diagnose different stages of cancers (Cervical cancer).

Biomarker	Normal	CIN1	CIN2	CIN3	I	II	III	IV	Ref
MiB-1	10%>	>10%	-	-	>71%	>I	>II,I	High	[140]
CDC6	90%	38%	-	24%	-	-	70%	-	[141]
CD44	-	-	-	-	High	<I	<I,II	<I,II,III	[142]
CK7	Positive	-	-	-	-	-	negative	Negative	[143]
hMSH2	-	-	-	-	-	-	54.3%	-	[71]
Ki-67	20%>	22.6%	82.1%	88.6%	-	-	100%	-	[144]
p16	>10%	10.4%	84.5%	90.5%	-	-	91.3%	-	[144]
SSC-Ag	-	-	-	-	24%	53%	75%	90%	[145]
MCM3	negative	4–6%	-	-	-	-	18%	-	[141]
p63	100%	100%	-	-	30%	-	-	10%	[146]
p53	<10%	10%	50%	71%	-	-	-	-	[146]

**Table 5:** Ki67 and p16 biomarkers enhance each other to improve cervical cancer sensitivity and specificity.

Biomarker	Sensitivity (%)	Specificity (%)
Ki67	95.2	86.7
P16	85.4	94.6
Ki67 + p16	94.8	93.2

## References

[1] Flepisi B.T., Bouic P., Sissolak G., Rosenkranz B. (2014). Biomarkers of HIV-associated Cancer. Biomark. Cancer.

[2] Casper C. (2011). The increasing burden of HIV-associated malignancies in resource-limited regions. Annu. Rev. Med..

[3] Sasco A.J., Jaquet A., Boidin E., Ekouevi D.K., Thouillot F., Lemabec T., Forstin M.A., Renaudier P., N’Dom P., Malvy D. (2010). The challenge of AIDS-related malignancies in sub-Saharan Africa. PLoS ONE.

[4] Chinula L., Moses A., Gopal S. (2017). HIV-associated malignancies in sub-Saharan Africa: Progress, challenges, and opportunities. Curr. Opin. HIV AIDS.

[5] Robbins H.A., Pfeiffer R.M., Shiels M.S., Li J., Hall H.I., Engels E.A. (2015). Excess cancers among HIV-infected people in the United States. J. Natl. Cancer Inst..

[6] Ambinder R.F., Bhatia K., Martinez-Maza O., Mitsuyasu R. (2010). Cancer biomarkers in HIV patients. Curr. Opin. HIV AIDS.

[7] Hathout Y., Brody E., Clemens P.R., Cripe L., DeLisle R.K., Furlong P., Gordish-Dressman H., Hache L., Henricson E., Hoffman E.P. (2015). Large-scale serum protein biomarker discovery in Duchenne muscular dystrophy. Proc. Natl. Acad. Sci. USA.

[8] McDonald W.H., Yates J.R. (2002). Shotgun proteomics and biomarker discovery. Dis. Markers.

[9] Verma M. (2012). Personalized medicine and cancer. J. Pers. Med..

[10] Altekruse S.F., Shiels M.S., Modur S.P., Land S.R., Crothers K.A., Kitahata M.M., Thorne J.E., Mathews W.C., Fernández-Santos D.M., Mayor A.M. (2018). Cancer burden attributable to cigarette smoking among HIV-infected people in North America. AIDS.

[11] Shiels M.S., Pfeiffer R.M., Gail M.H., Hall H.I., Li J., Chaturvedi A.K., Bhatia K., Uldrick T.S., Yarchoan R., Goedert J.J. (2011). Cancer burden in the HIV-infected population in the United States. J. Natl. Cancer Inst..

[12] Engels E.A., Pfeiffer R.M., Fraumeni J.F., Kasiske B.L., Israni A.K., Snyder J.J., Wolfe R.A., Goodrich N.P., Bayakly A.R., Clarke C.A. (2011). Spectrum of cancer risk among US solid organ transplant recipients. JAMA.

[13] Rubinstein P.G., Aboulafia D.M., Zloza A. (2014). Malignancies in HIV/AIDS: From epidemiology to therapeutic challenges. AIDS.

[14] Justice A.C., Erlandson K.M., Hunt P.W., Landay A., Miotti P., Tracy R.P. (2018). Can Biomarkers Advance HIV Research and Care in the Antiretroviral Therapy Era?. J. Infect. Dis..

[15] Chiao E.Y., Hartman C.M., El-Serag H.B., Giordano T.P. (2013). The impact of HIV viral control on the incidence of HIV-associated anal cancer. J. Acquir. Immune Defic. Syndr..

[16] Cheng L., Davison D.D., Adams J., Lopez-Beltran A., Wang L., Montironi R., Zhang S. (2014). Biomarkers in bladder cancer: Translational and clinical implications. Crit. Rev. Oncol. Hematol..

[17] Barreto L., Azambuja D., Morais J.C. (2012). Expression of immunohistochemical markers in patients with AIDS-related lymphoma. Braz. J. Infect. Dis..

[18] Borges A.H., Dubrow R., Silverberg M.J. (2014). Factors contributing to risk for cancer among HIV-infected individuals, and evidence that earlier combination antiretroviral therapy will alter this risk. Curr. Opin. HIV AIDS.

[19] Dryden-Peterson S., Medhin H., Kebabonye-Pusoentsi M., Seage G.R., Suneja G., Kayembe M.K., Mmalane M., Rebbeck T., Rider J.R., Essex M.J.P.O. (2015). Cancer incidence following expansion of HIV treatment in Botswana. PLoS ONE.

[20] Engels E.A. (2009). Non-AIDS-defining malignancies in HIV-infected persons: Etiologic puzzles, epidemiologic perils, prevention opportunities. AIDS.

[21] Yarchoan R., Uldrick T.S. (2018). HIV-Associated Cancers and Related Diseases. N. Engl. J. Med..

[22] Miller K.D., Goding Sauer A., Ortiz A.P., Fedewa S.A., Pinheiro P.S., Tortolero-Luna G., Martinez-Tyson D., Jemal A., Siegel R.L. (2018). Cancer statistics for hispanics/latinos. CA Cancer J. Clin..

[23] Engels E.A., Pfeiffer R.M., Goedert J.J., Virgo P., McNeel T.S., Scoppa S.M., Biggar R.J. (2006). Trends in cancer risk among people with AIDS in the United States 1980–2002. AIDS.

[24] Park L.S., Hernández-Ramírez R.U., Silverberg M.J., Crothers K., Dubrow R. (2016). Prevalence of non-HIV cancer risk factors in persons living with HIV/AIDS: A meta-analysis. AIDS.

[25] Pulitzer M. (2012). Molecular diagnosis of infection-related cancers in dermatopathology. Semin. Cutan. Med. Surg..

[26] Hull R., Mbele M., Makhafola T., Hicks C., Wang S.M., Reis R.M., Mehrotra R., Mkhize-Kwitshana Z., Kibiki G., Bates D.O. (2020). Cervical cancer in low and middle-income countries. Oncol. Lett..

[27] Chyla B., Daver N., Doyle K., McKeegan E., Huang X., Ruvolo V., Wang Z., Chen K., Souers A., Leverson J. (2018). Genetic Biomarkers of Sensitivity and Resistance to Venetoclax Monotherapy in Patients with Relapsed Acute Myeloid Leukemia. Am. J. Hematol..

[28] Banerjee J., Pradhan R., Gupta A., Kumar R., Sahu V., Upadhyay A.D., Chaterjee P., Dwivedi S., Dey S., Dey A.B. (2017). CDK4 in lung, and head and neck cancers in old age: Evaluation as a biomarker. Clin. Transl. Oncol..

[29] Sahutoglu T., Sakaci T., Hasbal N.B., Ahbap E., Kara E., Sumerkan M.C., Sevinc M., Akgol C., Koc Y., Basturk T. (2017). Serum VEGF-C levels as a candidate biomarker of hypervolemia in chronic kidney disease. Medicine.

[30] Heckman-Stoddard B.M. (2012). Oncology biomarkers: Discovery, validation, and clinical use. Semin. Oncol. Nurs..

[31] Waseem M., Ahmad M.K., Srivatava V.K., Rastogi N., Serajuddin M., Kumar S., Mishra D.P., Sankhwar S.N., Mahdi A.A. (2017). Evaluation of miR-711 as Novel Biomarker in Prostate Cancer Progression. Asian Pac. J. Cancer Prev. APJCP.

[32] Lesko L.J., Atkinson A.J. (2001). Use of biomarkers and surrogate endpoints in drug development and regulatory decision making: Criteria, validation, strategies. Annu. Rev. Pharmacol. Toxicol..

[33] Maisel A.S., Katz N., Hillege H.L., Shaw A., Zanco P., Bellomo R., Anand I., Anker S.D., Aspromonte N., Bagshaw S.M. (2011). Biomarkers in kidney and heart disease. Nephrol. Dial. Transplant..

[34] Alizadeh A.A., Eisen M.B., Davis R.E., Ma C., Lossos I.S., Rosenwald A., Boldrick J.C., Sabet H., Tran T., Yu X. (2000). Distinct types of diffuse large B-cell lymphoma identified by gene expression profiling. Nature.

[35] Cai Q., Verma S.C., Choi J.Y., Ma M., Robertson E.S. (2010). Kaposi’s sarcoma-associated herpesvirus inhibits interleukin-4-mediated STAT6 phosphorylation to regulate apoptosis and maintain latency. J. Virol..

[36] Cai Q., Verma S.C., Kumar P., Ma M., Robertson E.S. (2010). Hypoxia inactivates the VHL tumor suppressor through PIASy-mediated SUMO modification. PLoS ONE.

[37] Verma M. (2014). Molecular profiling and companion diagnostics: Where is personalized medicine in cancer heading?. Pers. Med..

[38] Verma S., Lu X., Ma S., Masel R.I., Kenis P.J. (2016). The effect of electrolyte composition on the electroreduction of CO_2_ to CO on Ag based gas diffusion electrodes. Phys. Chem. Chem. Phys..

[39] Vendrame E., Hussain S.K., Breen E.C., Magpantay L.I., Widney D.P., Jacobson L.P., Variakojis D., Knowlton E.R., Bream J.H., Ambinder R.F. (2014). Serum levels of cytokines and biomarkers for inflammation and immune activation, and HIV-associated non-Hodgkin B-cell lymphoma risk. Cancer Epidemiol. Biomark. Prev..

[40] Selvi K., Badhe B.A., Papa D., Ganesh R.N. (2014). Role of p16, CK17, p63, and human papillomavirus in diagnosis of cervical intraepithelial neoplasia and distinction from its mimics. Int. J. Surg. Pathol..

[41] Izadi-Mood N., Sarmadi S., Eftekhar Z., Jahanteegh H.A., Sanii S. (2014). Immunohistochemical expression of p16 and HPV L1 capsid proteins as predictive markers in cervical lesions. Arch. Gynecol. Obstet..

[42] Soliman P.T., Langley G., Munsell M.F., Vaniya H.A., Frumovitz M., Ramirez P.T. (2013). Analgesic and antiemetic requirements after minimally invasive surgery for early cervical cancer: A comparison between laparoscopy and robotic surgery. Ann. Surg. Oncol..

[43] Madhumati G., Kavita S., Anju M., Uma S., Raj M. (2012). Immunohistochemical Expression of Cell Proliferating Nuclear Antigen (PCNA) and p53 Protein in Cervical Cancer. J. Obstet. Gynaecol. India.

[44] Ren C., Zhu Y., Yang L., Zhang X., Liu L., Wang Z., Jiang D. (2019). Prognostic and diagnostic validity of p16/Ki-67, HPV E6/E7 mRNA, and HPV DNA in women with ASCUS: A follow-up study. Virol. J..

[45] Ren C., Zhu Y., Yang L., Zhang X., Liu L., Ren C. (2018). Diagnostic performance of HPV E6/E7 mRNA assay for detection of cervical high-grade intraepithelial neoplasia and cancer among women with ASCUS Papanicolaou smears. Arch. Gynecol. Obstet..

[46] Derbie A., Mekonnen D., Woldeamanuel Y., Van Ostade X., Abebe T. (2020). HPV E6/E7 mRNA test for the detection of high grade cervical intraepithelial neoplasia (CIN2+): A systematic review. Infect. Agents Cancer.

[47] Campbell L.M., Pitta D.R., De Assis A.M., Derchain S.F., Campos E.A., Sarian L.O. (2013). Retrieval of HPV oncogenes E6 and E7 mRNA from cervical specimens using a manual open technology protocol. SpringerPlus.

[48] Hu Z., Ma D. (2018). The precision prevention and therapy of HPV-related cervical cancer: New concepts and clinical implications. Cancer Med..

[49] Basu P., Banerjee D., Mittal S., Dutta S., Ghosh I., Chowdhury N., Abraham P., Chandna P., Ratnam S. (2016). Sensitivity of APTIMA HPV E6/E7 mRNA test in comparison with hybrid capture 2 HPV DNA test for detection of high risk oncogenic human papillomavirus in 396 biopsy confirmed cervical cancers. J. Med. Virol..

[50] Ratnam S., Coutlee F., Fontaine D., Bentley J., Escott N., Ghatage P., Gadag V., Holloway G., Bartellas E., Kum N. (2011). Aptima HPV E6/E7 mRNA test is as sensitive as Hybrid Capture 2 Assay but more specific at detecting cervical precancer and cancer. J. Clin. Microbiol..

[51] Coolen M., Katz S., Bally-Cuif L. (2013). miR-9: A versatile regulator of neurogenesis. Front. Cell. Neurosci..

[52] Xu W., Xu M., Wang L., Zhou W., Xiang R., Shi Y., Zhang Y., Piao Y. (2019). Integrative analysis of DNA methylation and gene expression identified cervical cancer-specific diagnostic biomarkers. Signal Transduct. Target. Ther..

[53] Dashti N., Mahmoudi M., Gharibdoost F., Kavosi H., Rezaei R., Imeni V., Jamshidi A., Aslani S., Mostafaei S., Vodjgani M. (2018). Evaluation of ITGB2 (CD18) and SELL (CD62L) genes expression and methylation of ITGB2 promoter region in patients with systemic sclerosis. Rheumatol. Int..

[54] Nyman J., Mercke C., Lindström J. (1993). Prognostic factors for local control and survival of cancer of the oral tongue. A retrospective analysis of 230 cases in western Sweden. Acta Oncol..

[55] Carozzi F., Gillio-Tos A., Confortini M., Del Mistro A., Sani C., De Marco L., Girlando S., Rosso S., Naldoni C., Dalla Palma P. (2013). Risk of high-grade cervical intraepithelial neoplasia during follow-up in HPV-positive women according to baseline p16-INK4A results: A prospective analysis of a nested substudy of the NTCC randomised controlled trial. Lancet Oncol..

[56] Cecchini S., Carozzi F., Confortini M., Zappa M., Ciatto S. (2004). Persistent human papilloma virus infection as an indicator of risk of recurrence of high-grade cervical intraepithelial neoplasia treated by the loop electrosurgical excision procedure. Tumori J..

[57] Martens J.E., Arends J., Van der Linden P.J., De Boer B.A., Helmerhorst T.J. (2004). Cytokeratin 17 and p63 are markers of the HPV target cell, the cervical stem cell. Anticancer Res..

[58] Ikeda K., Tate G., Suzuki T., Mitsuya T. (2008). Coordinate expression of cytokeratin 8 and cytokeratin 17 immunohistochemical staining in cervical intraepithelial neoplasia and cervical squamous cell carcinoma: An immunohistochemical analysis and review of the literature. Gynecol. Oncol..

[59] Garima, Pandey S., Pandey L.K., Saxena A.K., Patel N. (2016). The Role of p53 Gene in Cervical Carcinogenesis. J. Obstet. Gynaecol. India.

[60] Romus I., Triningsih F.E., Mangunsudirdjo S., Harijadi A. (2013). Clinicopathology significance of p53 and p63 expression in Indonesian cervical squamous cell carcinomas. Asian Pac. J. Cancer Prev..

[61] Zajkowska M., Zbucka-Krętowska M., Sidorkiewicz I., Lubowicka E., Gacuta E., Szmitkowski M., Chrostek L., Ławicki S. (2018). Plasma levels and diagnostic utility of macrophage-colony stimulating factor, matrix metalloproteinase-9 and tissue inhibitor of metalloproteinase-1 as tumor markers in cervical cancer patients. Tumour Biol..

[62] Ceci C., Atzori M.G., Lacal P.M., Graziani G. (2020). Role of VEGFs/VEGFR-1 Signaling and its Inhibition in Modulating Tumor Invasion: Experimental Evidence in Different Metastatic Cancer Models. Int. J. Mol. Sci..

[63] Mishra A., Verma M. (2010). Cancer biomarkers: Are we ready for the prime time?. Cancers.

[64] Roncaglia M.T., Fregnani J.H., Tacla M., De Campos S.G., Caiaffa H.H., Ab’saber A., Da Motta E.V., Alves V.A., Baracat E.C., Longatto Filho A. (2013). Characterization of p16 and E6 HPV-related proteins in uterine cervix high-grade lesions of patients treated by conization with large loop excision. Oncol. Lett..

[65] Sari Aslani F., Safaei A., Pourjabali M., Momtahan M. (2013). Evaluation of Ki67, p16 and CK17 Markers in Differentiating Cervical Intraepithelial Neoplasia and Benign Lesions. Iran. J. Med. Sci..

[66] Iaconis L., Hyjek E., Ellenson L.H., Pirog E.C. (2007). p16 and Ki-67 immunostaining in atypical immature squamous metaplasia of the uterine cervix: Correlation with human papillomavirus detection. Arch. Pathol. Lab. Med..

[67] Regauer S., Reich O. (2007). CK17 and p16 expression patterns distinguish (atypical) immature squamous metaplasia from high-grade cervical intraepithelial neoplasia (CIN III). Histopathology.

[68] Ishimi Y., Okayasu I., Kato C., Kwon H.J., Kimura H., Yamada K., Song S.Y. (2003). Enhanced expression of Mcm proteins in cancer cells derived from uterine cervix. Eur. J. Biochem..

[69] Das M., Prasad S.B., Yadav S.S., Govardhan H.B., Pandey L.K., Singh S., Pradhan S., Narayan G. (2013). Over expression of minichromosome maintenance genes is clinically correlated to cervical carcinogenesis. PLoS ONE.

[70] Bonds L., Baker P., Gup C., Shroyer K.R. (2002). Immunohistochemical localization of cdc6 in squamous and glandular neoplasia of the uterine cervix. Arch. Pathol. Lab. Med..

[71] Murphy N., Ring M., Heffron C.C., Martin C.M., McGuinness E., Sheils O., O’Leary J.J. (2005). Quantitation of CDC6 and MCM5 mRNA in cervical intraepithelial neoplasia and invasive squamous cell carcinoma of the cervix. Mod. Pathol..

[72] Cheng Q., Lau W.M., Chew S.H., Ho T.H., Tay S.K., Hui K.M. (2002). Identification of molecular markers for the early detection of human squamous cell carcinoma of the uterine cervix. Br. J. Cancer.

[73] Portari E.A., Russomano F.B., de Camargo M.J., Machado Gayer C.R., da Rocha Guillobel H.C., Santos-Rebouças C.B., Brito Macedo J.M. (2013). Immunohistochemical expression of cyclin D1, p16Ink4a, p21WAF1, and Ki-67 correlates with the severity of cervical neoplasia. Int. J. Gynecol. Pathol..

[74] Branca M., Ciotti M., Giorgi C., Santini D., Di Bonito L., Costa S., Benedetto A., Bonifacio D., Di Bonito P., Paba P. (2007). Up-regulation of proliferating cell nuclear antigen (PCNA) is closely associated with high-risk human papillomavirus (HPV) and progression of cervical intraepithelial neoplasia (CIN), but does not predict disease outcome in cervical cancer. Eur. J. Obstet. Gynecol. Reprod. Biol..

[75] Goel M.M., Mehrotra A. (2013). Immunohistochemical expression of MIB-1 and PCNA in precancerous and cancerous lesions of uterine cervix. Indian J. Cancer.

[76] Zhou Y., Xu Q., Ling B., Xiao W., Liu P. (2011). Reduced expression of ΔΝp63α in cervical squamous cell carcinoma. Clin. Investig. Med. Med. Clin. Exp..

[77] Speiser P., Wanner C., Tempfer C., Mittelböck M., Hanzal E., Bancher-Todesca D., Gitsch G., Reinthaller A., Kainz C. (1997). CD44 is an independent prognostic factor in early-stage cervical cancer. Int. J. Cancer.

[78] Emmanuel B., Anderson W.F. (2012). Non-Hodgkin lymphoma in early life. J. Natl. Cancer Inst..

[79] Sangle N.A., Agarwal A.M., Smock K.J., Leavitt M.O., Warnke R., Bahler D., Perkins S.L. (2011). Diffuse large B-cell lymphoma with aberrant expression of the T-cell antigens CD2 and CD7. Appl. Immunohistochem. Mol. Morphol..

[80] Kim M.K., Bae S.H., Bae Y.K., Kum Y.S., Ryoo H.M., Cho H.S., Lee K.H., Koh S.A., Lee H.Y., Yun S.Y. (2011). Biological characterization of nodal versus extranodal presentation of diffuse large B-Cell lymphoma using immunohistochemistry. Clin. Lymphoma Myeloma Leuk..

[81] Pörtner L.M., Schönberg K., Hejazi M., Brünnert D., Neumann F., Galonska L., Reusch U., Little M., Haas R., Uhrberg M. (2012). T and NK cells of B cell NHL patients exert cytotoxicity against lymphoma cells following binding of bispecific tetravalent antibody CD19 × CD3 or CD19 × CD16. Cancer Immunol. Immunother..

[82] Davenport R.J. (2005). Will we find biomarkers of aging?. Sci. Aging Knowl. Environ. SAGE KE.

[83] Nyman H., Adde M., Karjalainen-Lindsberg M.L., Taskinen M., Berglund M., Amini R.M., Blomqvist C., Enblad G., Leppä S. (2007). Prognostic impact of immunohistochemically defined germinal center phenotype in diffuse large B-cell lymphoma patients treated with immunochemotherapy. Blood.

[84] Visco C., Li Y., Xu-Monette Z.Y., Miranda R.N., Green T.M., Li Y., Tzankov A., Wen W., Liu W.M., Kahl B.S. (2012). Comprehensive gene expression profiling and immunohistochemical studies support application of immunophenotypic algorithm for molecular subtype classification in diffuse large B-cell lymphoma: A report from the International DLBCL Rituximab-CHOP Consortium Program Study. Leukemia.

[85] Navarro J.T., Ribera J.M., Oriol A., Vaquero M., Romeu J., Batlle M., Gómez J., Millá F., Feliu E. (1998). International prognostic index is the best prognostic factor for survival in patients with AIDS-related non-Hodgkin’s lymphoma treated with CHOP. A multivariate study of 46 patients. Haematologica.

[86] Leong T.L., Marini K.D., Rossello F.J., Jayasekara S.N., Russell P.A., Prodanovic Z., Kumar B., Ganju V., Alamgeer M., Irving L.B. (2014). Genomic characterisation of small cell lung cancer patient-derived xenografts generated from endobronchial ultrasound-guided transbronchial needle aspiration specimens. PLoS ONE.

[87] Steinfort D.P., Johnson D.F., Connell T.G., Irving L.B. (2009). Endobronchial ultrasound-guided biopsy in the evaluation of intrathoracic lymphadenopathy in suspected tuberculosis: A minimally invasive technique with a high diagnostic yield. J. Infect..

[88] Kaplan L.D. (2012). HIV-associated lymphoma. Pract. Res. Clin. Haematol..

[89] Kaplan L.D. (2012). Management of HIV-associated Hodgkin lymphoma: How far we have come. J. Clin. Oncol..

[90] Tang Y.L., Zhou Y., Cheng L.L., Su Y.Z., Wang C.B. (2017). BCL2/Ki-67 index predict survival in germinal center B-cell-like diffuse large B-cell lymphoma. Oncol. Lett..

[91] Weissman D., Dybul M., Daucher M.B., Davey R.T., Walker R.E., Kovacs J.A. (2000). Interleukin-2 up-regulates expression of the human immunodeficiency virus fusion coreceptor CCR5 by CD4+ lymphocytes in vivo. J. Infect. Dis..

[92] Chen X., Cheng L., Jia X., Zeng Y., Yao S., Lv Z., Qin D., Fang X., Lei Y., Lu C. (2009). Human immunodeficiency virus type 1 Tat accelerates Kaposi sarcoma-associated herpesvirus Kaposin A-mediated tumorigenesis of transformed fibroblasts in vitro as well as in nude and immunocompetent mice. Neoplasia.

[93] Suzuki K., Terui Y., Nishimura N., Mishima Y., Sakajiri S., Yokoyama M., Takahashi S., Tsuyama N., Takeuchi K., Hatake K. (2013). Prognostic value of C-reactive protein, lactase dehydrogenase and anemia in recurrent or refractory aggressive lymphoma. Jpn. J. Clin. Oncol..

[94] De Roos A.J., Mirick D.K., Edlefsen K.L., LaCroix A.Z., Kopecky K.J., Madeleine M.M., Magpantay L., Martínez-Maza O. (2012). Markers of B-cell activation in relation to risk of non-Hodgkin lymphoma. Cancer Res..

[95] Tedeschi R., Bortolin M.T., Bidoli E., Zanussi S., Pratesi C., Vaccher E., Tirelli U., De Paoli P. (2012). Assessment of immunovirological features in HIV related non-Hodgkin lymphoma patients and their impact on outcome. J. Clin. Virol..

[96] Long E., Ilie M., Hofman V., Havet K., Selva E., Butori C., Lacour J.P., Nelson A.M., Cathomas G., Hofman P. (2009). LANA-1, Bcl-2, Mcl-1 and HIF-1alpha protein expression in HIV-associated Kaposi sarcoma. Virchows Arch..

[97] Ojala P.M., Tiainen M., Salven P., Veikkola T., Castaños-Vélez E., Sarid R., Biberfeld P., Mäkelä T.P. (1999). Kaposi’s sarcoma-associated herpesvirus-encoded v-cyclin triggers apoptosis in cells with high levels of cyclin-dependent kinase 6. Cancer Res..

[98] Pantanowitz L., Schwartz E.J., Dezube B.J., Kohler S., Dorfman R.F., Tahan S.R. (2005). C-Kit (CD117) expression in AIDS-related, classic, and African endemic Kaposi sarcoma. Appl. Immunohistochem. Mol. Morphol..

[99] Schwartz E.J., Dorfman R.F., Kohler S. (2003). Human herpesvirus-8 latent nuclear antigen-1 expression in endemic Kaposi sarcoma: An immunohistochemical study of 16 cases. Am. J. Surg. Pathol..

[100] Milanovic N., Matkovic S., Ristic D., Jelic S., Petrovic M. (2012). Significance of tumor burden, vascular endothelial growth factor, lactate dehydrogenase and beta-2 microglobulin serum levels in advanced diffuse large B cell lymphoma. J. BUON.

[101] Bairey O., Bar-Natan M., Shpilberg O. (2013). Early death in patients diagnosed with non-Hodgkin’s lymphoma. Ann. Hematol..

[102] Chao C., Silverberg M.J., Martínez-Maza O., Chi M., Abrams D.I., Haque R., Zha H.D., McGuire M., Xu L., Said J. (2012). Epstein-Barr virus infection and expression of B-cell oncogenic markers in HIV-related diffuse large B-cell Lymphoma. Clin. Cancer Res..

[103] Zinzani P.L., Dirnhofer S., Sabattini E., Alinari L., Piccaluga P.P., Stefoni V., Tani M., Musuraca G., Marchi E., Falini B. (2005). Identification of outcome predictors in diffuse large B-cell lymphoma. Immunohistochemical profiling of homogeneously treated de novo tumors with nodal presentation on tissue micro-arrays. Haematologica.

[104] Miles R.R., Arnold S., Cairo M.S. (2012). Risk factors and treatment of childhood and adolescent Burkitt lymphoma/leukaemia. Br. J. Haematol..

[105] De Mello C.A., De Andrade V.P., De Lima V.C., Carvalho A.L., Soares F.A. (2011). Prognostic impact of MUM1 expression by immunohistochemistry on primary mediastinal large B-cell lymphoma. Leuk. Lymphoma.

[106] Mead G.M., Barrans S.L., Qian W., Walewski J., Radford J.A., Wolf M., Clawson S.M., Stenning S.P., Yule C.L., Jack A.S. (2008). A prospective clinicopathologic study of dose-modified CODOX-M/IVAC in patients with sporadic Burkitt lymphoma defined using cytogenetic and immunophenotypic criteria (MRC/NCRI LY10 trial). Blood.

[107] Ladanyi M., Offit K., Jhanwar S.C., Filippa D.A., Chaganti R.S. (1991). MYC rearrangement and translocations involving band 8q24 in diffuse large cell lymphomas. Blood.

[108] Breen E.C., Hussain S.K., Magpantay L., Jacobson L.P., Detels R., Rabkin C.S., Kaslow R.A., Variakojis D., Bream J.H., Rinaldo C.R.J.C.E. (2011). B-cell stimulatory cytokines and markers of immune activation are elevated several years prior to the diagnosis of systemic AIDS–associated non-hodgkin B-Cell lymphoma. Cancer Epidemiol. Prev. Biomark..

[109] Masood R., Zhang Y., Bond M.W., Scadden D.T., Moudgil T., Law R.E., Kaplan M.H., Jung B., Espina B.M., Lunardi-Iskandar Y. (1995). Interleukin-10 is an autocrine growth factor for acquired immunodeficiency syndrome-related B-cell lymphoma. Blood.

[110] Nakayama S., Yokote T., Hirata Y., Akioka T., Miyoshi T., Hiraoka N., Iwaki K., Takayama A., Nishiwaki U., Masuda Y. (2014). TNF-α expression in tumor cells as a novel prognostic marker for diffuse large B-cell lymphoma, not otherwise specified. Am. J. Surg. Pathol..

[111] Landgren O., Goedert J.J., Rabkin C.S., Wilson W.H., Dunleavy K., Kyle R.A., Katzmann J.A., Rajkumar S.V., Engels E.A. (2010). Circulating serum free light chains as predictive markers of AIDS-related lymphoma. J. Clin. Oncol..

[112] Yu B., Zhou X., Li B., Xiao X., Yan S., Shi D. (2011). FOXP1 expression and its clinicopathologic significance in nodal and extranodal diffuse large B-cell lymphoma. Ann. Hematol..

[113] Ceccarelli M., Facciolà A., Taibi R., Pellicanò G.F., Nunnari G., Venanzi Rullo E. (2019). The treatment of Kaposi’s sarcoma: Present and future options, a review of the literature. Eur. Rev. Med. Pharmacol. Sci..

[114] Pereira P.F., Cuzzi T., Galhardo M.C. (2013). Immunohistochemical detection of the latent nuclear antigen-1 of the human herpesvirus type 8 to differentiate cutaneous epidemic Kaposi sarcoma and its histological simulators. An. Bras. Dermatol..

[115] Pantanowitz L., Dezube B.J., Pinkus G.S., Tahan S.R. (2004). Histological characterization of regression in acquired immunodeficiency syndrome-related Kaposi’s sarcoma. J. Cutan. Pathol..

[116] Cheuk W., Wong K.O., Wong C.S., Dinkel J.E., Ben-Dor D., Chan J.K. (2004). Immunostaining for human herpesvirus 8 latent nuclear antigen-1 helps distinguish Kaposi sarcoma from its mimickers. Am. J. Clin. Pathol..

[117] Horenstein M.G., Cesarman E., Wang X., Linkov I., Prieto V.G., Louie D.C. (1997). Cyclin D1 and retinoblastoma protein expression in Kaposi’s sarcoma. J. Cutan. Pathol..

[118] Hong A., Davies S., Stevens G., Lee C.S. (2004). Cyclin D1 overexpression in AIDS-related and classic Kaposi sarcoma. Appl. Immunohistochem. Mol. Morphol..

[119] Moses A.V., Jarvis M.A., Raggo C., Bell Y.C., Ruhl R., Luukkonen B.G., Griffith D.J., Wait C.L., Druker B.J., Heinrich M.C. (2002). Kaposi’s sarcoma-associated herpesvirus-induced upregulation of the c-kit proto-oncogene, as identified by gene expression profiling, is essential for the transformation of endothelial cells. J. Virol..

[120] Cai X., Lu S., Zhang Z., Gonzalez C.M., Damania B., Cullen B.R. (2005). Kaposi’s sarcoma-associated herpesvirus expresses an array of viral microRNAs in latently infected cells. Proc. Natl. Acad. Sci. USA.

[121] Sakakibara S., Pise-Masison C.A., Brady J.N., Tosato G. (2009). Gene regulation and functional alterations induced by Kaposi’s sarcoma-associated herpesvirus-encoded ORFK13/vFLIP in endothelial cells. J. Virol..

[122] Ballon G., Chen K., Perez R., Tam W., Cesarman E. (2011). Kaposi sarcoma herpesvirus (KSHV) vFLIP oncoprotein induces B cell transdifferentiation and tumorigenesis in mice. J. Clin. Investig..

[123] Kennedy M.M., Biddolph S., Lucas S.B., Howells D.D., Picton S., McGee J.O., Silva I., Uhlmann V., Luttich K., O’Leary J.J. (1999). Cyclin D1 expression and HHV8 in Kaposi sarcoma. J. Clin. Pathol..

[124] Koopal S., Furuhjelm J.H., Järviluoma A., Jäämaa S., Pyakurel P., Pussinen C., Wirzenius M., Biberfeld P., Alitalo K., Laiho M. (2007). Viral oncogene-induced DNA damage response is activated in Kaposi sarcoma tumorigenesis. PLoS Pathog..

[125] Friborg J., Kong W., Hottiger M.O., Nabel G.J. (1999). p53 inhibition by the LANA protein of KSHV protects against cell death. Nature.

[126] Si H., Robertson E.S. (2006). Kaposi’s sarcoma-associated herpesvirus-encoded latency-associated nuclear antigen induces chromosomal instability through inhibition of p53 function. J. Virol..

[127] Kahn H.J., Bailey D., Marks A. (2002). Monoclonal antibody D2-40, a new marker of lymphatic endothelium, reacts with Kaposi’s sarcoma and a subset of angiosarcomas. Mod. Pathol..

[128] Nagata N., Igari T., Shimbo T., Sekine K., Akiyama J., Hamada Y., Yazaki H., Ohmagari N., Teruya K., Oka S. (2013). Diagnostic value of endothelial markers and HHV-8 staining in gastrointestinal Kaposi sarcoma and its difference in endoscopic tumor staging. World J. Gastroenterol..

[129] Rosado F.G., Itani D.M., Coffin C.M., Cates J.M. (2012). Utility of immunohistochemical staining with FLI1, D2-40, CD31, and CD34 in the diagnosis of acquired immunodeficiency syndrome-related and non-acquired immunodeficiency syndrome-related Kaposi sarcoma. Arch. Pathol. Lab. Med..

[130] Russell Jones R., Orchard G., Zelger B., Wilson Jones E. (1995). Immunostaining for CD31 and CD34 in Kaposi sarcoma. J. Clin. Pathol..

[131] Aoki Y., Yarchoan R., Wyvill K., Okamoto S., Little R.F., Tosato G. (2001). Detection of viral interleukin-6 in Kaposi sarcoma-associated herpesvirus-linked disorders. Blood.

[132] Guo Z., Gillam E.M., Ohmori S., Tukey R.H., Guengerich F.P. (1994). Expression of modified human cytochrome P450 1A1 in Escherichia coli: Effects of 5′ substitution, stabilization, purification, spectral characterization, and catalytic properties. Arch. Biochem. Biophys..

[133] Faris M., Ensoli B., Kokot N., Nel A.E. (1998). Inflammatory cytokines induce the expression of basic fibroblast growth factor (bFGF) isoforms required for the growth of Kaposi’s sarcoma and endothelial cells through the activation of AP-1 response elements in the bFGF promoter. AIDS.

[134] Simonart T., Van Vooren J.P. (2002). Interleukin-1 beta increases the BCL-2/BAX ratio in Kaposi’s sarcoma cells. Cytokine.

[135] Cai J., Gill P.S., Masood R., Chandrasoma P., Jung B., Law R.E., Radka S.F. (1994). Oncostatin-M is an autocrine growth factor in Kaposi’s sarcoma. Am. J. Pathol..

[136] Amaral M.C., Miles S., Kumar G., Nel A.E. (1993). Oncostatin-M stimulates tyrosine protein phosphorylation in parallel with the activation of p42MAPK/ERK-2 in Kaposi’s cells. Evidence that this pathway is important in Kaposi cell growth. J. Clin. Investig..

[137] Chaturvedi A.K., Mbulaiteye S.M., Engels E.A. (2008). Underestimation of relative risks by standardized incidence ratios for AIDS-related cancers. Ann. Epidemiol..

[138] Shi Q., Xu L., Yang R., Meng Y., Qiu L. (2019). Ki-67 and P16 proteins in cervical cancer and precancerous lesions of young women and the diagnostic value for cervical cancer and precancerous lesions. Oncol. Lett..

[139] Schim van der Loeff M.F., Mooij S.H., Richel O., de Vries H.J., Prins J.M. (2014). HPV and anal cancer in HIV-infected individuals: A review. Curr. HIV/AIDS Rep..

[140] Mittal K. (1999). Utility of proliferation-associated marker MIB-1 in evaluating lesions of the uterine cervix. Adv. Anat. Pathol..

[141] Murphy N., Ring M., Heffron C.C., King B., Killalea A.G., Hughes C., Martin C.M., McGuinness E., Sheils O., O’Leary J.J. (2005). p16INK4A, CDC6, and MCM5: Predictive biomarkers in cervical preinvasive neoplasia and cervical cancer. J. Clin. Pathol..

[142] Chokchaichamnankit D., Watcharatanyatip K., Subhasitanont P., Weeraphan C., Keeratichamroen S., Sritana N., Kantathavorn N., Diskul-Na-Ayudthaya P., Saharat K., Chantaraamporn J. (2019). Urinary biomarkers for the diagnosis of cervical cancer by quantitative label-free mass spectrometry analysis. Oncol. Lett..

[143] Hashiguchi M., Masuda M., Kai K., Nakao Y., Kawaguchi A., Yokoyama M., Aishima S. (2019). Decreased cytokeratin 7 expression correlates with the progression of cervical squamous cell carcinoma and poor patient outcomes. J. Obstet. Gynaecol. Res..

[144] Kanthiya K., Khunnarong J., Tangjitgamol S., Puripat N., Tanvanich S. (2016). Expression of the p16 and Ki67 in Cervical Squamous Intraepithelial Lesions and Cancer. Asian Pac. J. Cancer Prev..

[145] Duk J.M., Groenier K.H., de Bruijn H.W., Hollema H., ten Hoor K.A., van der Zee A.G., Aalders J.G. (1996). Pretreatment serum squamous cell carcinoma antigen: A newly identified prognostic factor in early-stage cervical carcinoma. J. Clin. Oncol..

[146] Mitildzans A., Arechvo A., Rezeberga D., Isajevs S. (2017). Expression of p63, p53 and Ki-67 in Patients with Cervical Intraepithelial Neoplasia. Turk Patoloji Derg..

[147] Hessol N.A., Martínez-Maza O., Levine A.M., Morris A., Margolick J.B., Cohen M.H., Jacobson L.P., Seaberg E.C. (2015). Lung cancer incidence and survival among HIV-infected and uninfected women and men. AIDS.

[148] Thorsson V., Gibbs D.L., Brown S.D., Wolf D., Bortone D.S., Yang T.-H.O., Porta-Pardo E., Gao G.F., Plaisier C.L., Eddy J.A. (2019). The immune landscape of cancer. Immunity.

[149] Gopal S., Achenbach C.J., Yanik E.L., Dittmer D.P., Eron J.J., Engels E.A. (2014). Moving forward in HIV-associated cancer. J. Clin. Oncol..

